# Alpha fetoprotein promotes polarization of macrophages towards M2-like phenotype and inhibits macrophages to phagocytize hepatoma cells

**DOI:** 10.3389/fimmu.2023.1081572

**Published:** 2023-02-23

**Authors:** Minni Zhang, Kun Liu, Qiuyue Zhang, Junnv Xu, Jinchen Liu, Haifeng Lin, Bo Lin, Mingyue Zhu, Mengsen Li

**Affiliations:** ^1^ Hainan Provincial Key Laboratory of Carcinogenesis and Intervention, Hainan Medical College, Hiakou, Hainan, China; ^2^ Department of Medical Oncology, Second Affiliated Hospital, Hainan Medical College, Haikou, Hainan, China; ^3^ Institution of Tumor, Hainan Medical College, Hiakou, Hainan, China

**Keywords:** alpha-fetoprotein, macrophage polarization, PI3K/AKT signaling, macrophage phagocytosis, immune escape

## Abstract

Alpha-fetoprotein(AFP) is a cancer biomarker for the diagnosis of hepatocellular carcinoma(HCC); however, its role in macrophage polarization and phagocytosis remains unclear. In the present study, we explored the correlation between AFP regulation of macrophage function and the possible regulatory mechanisms. Human mononuclear leukemia cells (THP-1) and monocytes from healthy donors were used to analyze the effect of AFP on the macrophages’ phenotype and phagocytosis. THP-1 cells and healthy human donor-derived monocytes were polarized into M0 macrophages induced by phorbol ester (PMA), and M0 macrophages were polarized into M1 macrophages induced by lipopolysaccharide(LPS) and interferon-γ(IFN-γ). Interleukin-4(IL-4) and interleukin-13(IL-13) were used to induce M0 macrophage polarization into M2 macrophages. Tumor-derived AFP(tAFP) stimulated M0 macrophage polarization into M2 macrophages and inhibited M1 macrophages to phagocytize HCC cells. The role of AFP in promoting macrophage polarization into M2 macrophages and inhibiting the M1 macrophages to phagocytize HCC cells may be involved in activating the PI3K/Akt signaling pathway. AFP could also enhanced the migration ability of macrophages and inhibited the apoptosis of HCC cells when co-cultured with M1-like macrophages. AFP is a pivotal cytokine that inhibits macrophages to phagocytize HCC cells.

## 1 Introduction

The incidence and mortality of hepatocellular carcinoma (HCC) is increasing annually. The characteristics of high recurrence, easy metastasis, and a high degree of malignancy lead to high mortality, as well as great obstacles in improving the efficacy and survival rate (1). Chronic hepatitis virus infection, biliary tract diseases, long-term alcoholism, aflatoxin exposure, and drug use are important risk factors for the development of liver cancer (2, 3). Liver fibrosis and cirrhosis caused by hepatitis B virus(HBV) infection are the most important risk factors in China. In the case of cirrhosis, gene mutation, epigenetic dysregulation and abnormal molecular signal transduction are important causes of hepatocyte carcinogenesis (4). Alpha-fetoprotein (AFP), a single-chain serum glycoprotein belonging to the albumin family, is mainly synthesized by the fetal liver and yolk sac during embryonic development, and its concentration in serum decreases rapidly a few months after birth. Therefore, the concentration of AFP levels in normal adults is very low (<20 ng/mL) (5). Abnormal elevation of AFP is common in patients with chronic or active hepatitis, liver cirrhosis, liver cancer, genital tumors, and pregnancy, and approximately 70% of patients with liver cancer have elevated serum AFP levels. In clinical practice, AFP is regarded as a specific tumor biomarker for the screening and diagnosis of primary liver cancer, and it plays an important role in judging the degree of malignancy, evaluation of efficacy, detection of recurrence after surgery, liver transplantation, and guidance for clinical medication (6).

Tumor-associated macrophages (TAMs) in the tumor microenvironment (TME) can promote tumor development, invasion, metastasis, and recurrence (7). Generally, TAMs are M2 macrophages with phenotypic characteristics. Macrophages are mainly differentiated from circulating monocyte precursors and are characterized by high heterogeneity and functional diversity. The polarization of macrophages is an important factor affecting its anti-tumor activity (8), according to secreted cytokines and functions, macrophages can be divided into classical activated macrophages (M1 type) and alternatively activated macrophages (M2 type) (9, 10). M1-type macrophages are mainly induced and activated by lipopolysaccharide(LPS) and interferon-γ (IFN-γ). M1 macrophages can secrete proinflammatory cytokines, such as interleukin(IL)-12, IL-6, IL-1β, IL-23, and tumor necrosis factor-α(TNF-α), and also produce chemokines, such as CXCL9 and CXCL10. High expression of co-stimulatory molecules, such as CD86 and CD80, can also participate in inflammation by producing nitric oxide(NO), reactive oxygen species(ROS), and inducible nitric oxide synthase(iNOS) and by mediating the Th1-type cell immune response. It is mainly involved in the positive immune response through antigen presentation, and has strong phagocytosis and anti-tumor activity, thus exerting an immune surveillance function (11–14). M2-type macrophages are primarily induced and activated by IL-4, IL-13, and IL-10. The secretion of the inflammatory cytokines IL-10, transforming growth factor(TGF-β), and Arginase-1(Arg-1) and the expression of high levels of scavenger receptor (CD163), mannose receptor(CD206), CCL-17, CCL-22, and other chemokines downregulate the immune response. It mainly mediates the Th2-type immune response and plays a key anti-inflammatory role by accelerating tumor cells activation, invasion, angiogenesis, tissue remodeling, and inhibition of adaptive anti-tumor immunity (15–17).

Previous studies in our laboratory have shown that AFP could induce the expression of downstream target genes by activating the PI3K/Akt signaling pathway and regulating the growth, proliferation, invasion, metastasis, generation of stem cells, and other malignant behaviors of liver cancer cells (18–20). TAMs also play a key role in promoting HCC invasion and metastasis, immune escape, matrix remodeling, epithelial-mesenchymal transition(EMT), lymphangiogenesis and angiogenesis, and drug resistance (21). At present, there are few studies on whether AFP can regulate the PI3K/Akt signaling pathway to affect the polarization and phagocytosis of macrophages, reverse the macrophages phenotype, or reshape TAMs *in vivo*. In the present study, we investigated the role of AFP in the polarization and phagocytosis of macrophages, explored the inhibitory effect of AFP on cellular immunity, and identified a new function of AFP in stimulating HCC cells to escape the surveillance of immune cells.

## 2 Materials and methods

### 2.1 Cell lines and cell culture

The human mononuclear leukemia cell line, THP-1 was purchased from Wuhan Punoxai Life Technology Co., Ltd., while the human HCC cell lines Bel7402, HepG2 and HLE were purchased from Wuhan Boshude Bioengineering Co., Ltd. Cell resuscitation was performed using a UV lamp on a sterile ultraclean table. Cells were cultured in RPMI 1640 medium supplemented with 10% heat-inactivated fetal calf serum(FCS) and incubated at 37°C in a humidified atmosphere containing 5% CO_2_, as previously described (22, 23). Fresh medium (5 mL) was added to the new cell flask, the name of the cell was labeled with the name of the operator and the date, and then placed in the cell incubator for preheating. Cell status was observed, and the liquid was changed the next day.

### 2.2 Inducing macrophage polarization

THP-1 and cells were centrifuged at 800 RPM/min for 5 min, the supernatant was discarded, and the number of cells was adjusted to 1×10^6^/mL by adding an appropriate amount of fresh medium. Phorbol ester(PMA) was added to the cell suspension to a final concentration of 50 ng/mL, gently blown and mixed, and the cell suspension was seeded into a six-well plate at a volume of 2 mL per well and placed in an incubator under light. After treatment with PMA for 48 h, the cells turned from a suspension to adherent cells with protruding pseudopodia and were viewed under a microscope. THP-1 cells differentiated into M0 macrophages. M0 macrophages were washed twice with an appropriate amount of sterile PBS and cultured in fresh medium containing 50 ng/mL LPS+20 ng/mL IFN-γ for 24 h to obtain M1-type macrophages. Fresh medium containing 20 ng/mL IL-4+20 ng/mL IL-13 was added, and M0 macrophages were cultured for 72 h to obtain M2-type macrophages.

### 2.3 Lentiviral infection and screening of stable expression cell lines

For adherent Bel7402 cells, the cell number was diluted to 1.2×10^5^ cells/mL, and the cells were seeded into a 24-well plate at 500 μL/well. The culture was continued, and viral infection was performed when the degree of cell fusion reached 40%. For suspended THP-1 cells, The number of cells was diluted to 1×10^5^/mL, and 500 μL/well was seeded into a 24-well plate for direct viral infection. Then, 250 μL of fresh medium containing 1×Hitans GP or 1×Hitans GA was added, and the corresponding virus volume was converted according to the selected multiplicity of infection(MOI) gradient (10, 24–26) and added to fresh medium containing the viral infection booster solution. Cell culture plates were shaken using the crossing method. After 4 h, 250 μL of fresh medium containing the infection booster solution was added again for 15 h. The cells were washed twice with sterile PBS, and fresh virus-free medium was added. The efficiency of the viral infection was observed after 48 and 72 h. After the puromycin concentration was screened, cells in the blank group were seeded in a 24-well plate, and the culture medium was replaced with fresh medium containing puromycin after 24 h. The puromycin screening gradients was 0.6, 1.2, 1.8, 2.4, and 3.0 μg/mL. The fresh medium was replaced according to the cell state, and the minimum puromycin concentration that killed all cells in the blank group for 3-4 days was selected as the experimental concentration of infected lentiviral cell lines. To screen stable virus-infected cell lines, 72 h after lentiviral infection, the concentration of puromycin found in the pre-experiment was used to simultaneously screen the lentivirus-infected and blank groups. After the cells in the blank group died completely, the concentration of puromycin in the lentivirus-infected cells was reduced to 50% of the original concentration and the culture was maintained. After 3 days, the medium was replaced with fresh medium without puromycin, and the obtained cells were considered THP-1 stable cells.

### 2.4 Real-time polymerase chain reaction (PCR)

Total RNA extraction, RNA-free centrifuge tubes, EP tubes, PCR tubes, and pipetting nozzles were used in this experiment. The macrophages were washed twice with sterile PBS, the PBS was aspirated, an appropriate amount of ACCUTASE cell digestion solution was added, and the cells were shaken gently and placed in a cell incubator. When the cells gradually fell off the bottle, sterile PBS was added, the adherent cells were blown with a pipette gun, and the cell suspension was centrifuged at 1500 RPM/min for 10 min. The temperature of the water bath was maintained at 70°C. The supernatant was discarded and 300 μL of the lysate was added to the cell precipitate and gently aspirated using a pipette gun. Next, 300 μL of RNA diluent was added, mixed well, and placed in a water bath at 70°C for 3 min to improve the RNA yield. The procedure was as follows: 600 μL of RNA wash solution was added to the column, centrifuge at 12000 RPM/min for 1 min, the filtrate obtained in the collection tube was discarded, and 50 μL DNase I incubation solution was added to the middle of the adsorption membrane of each tube of the centrifugation column and incubated at room temperature for 15 min. Add 600 μL RNA solution, centrifuge at 12000 RPM/min for 1 min, discard the filtrate obtained in the collection tube, and repeat the above procedure twice. The concentration and purity of RNA were determined using an ultramicro spectrophotometer, and the RNA was stored at -80°C until further analysis. The mRNA levels of the target genes were detected by real-time PCR as previously described (27) and the primers were shown in **Table 1**.

**Table 1:** Primers for real-time polymerase chain reaction (PCR).

Gene name	primer sequences (5’ to 3’)
*GAPDH*	Forward: TGATGACATCAAGAAGGTGGTGAAG Reverse: TCCTTGGAGGCCATGTGGGCCAT
*CD86*	Forward: CTGCTCATCTATACACGGTTA Reverse: GGAAACGTCGTCAGTTCTGTG
*TNF-α*	Forward: TGGCCCAGGCAGTCAGA Reverse: GGTTTGCTACAACATGGGCTACA
*CD163*	Forward: TTTGTCAACTTGAGTCCCTTCAC Reverse: TCCCGCTACACTTGTTTTCAC
*Arg-1*	Forward: ACGGAAGAATCAGCCTGGTG Reverse: GTCCACGTCTCTCAAGCCAA

### 2.5 Western blotting analysis

To estimate the polarization of macrophages induced by PMA, lipopolysaccharide(LPS), interferon-γ(IFN-γ), IL-4, and IL-13, the expression of the markers of M1-type macrophages, CD86 and inducible nitric oxide synthase(iNOS), and the markers of M2-type macrophages, CD163 and IL-10, were detected by Western blotting. The influence of AFP on the expression of these marker proteins and PI3K/Akt signaling pathway-related proteins was analyzed in M0-type macrophages. M0 macrophages were infected with the AFP-expressed lentiviral vectors and treated with the PI3K inhibitor Ly294002 (final concentration:20 μM) for 48 h. The expression of these proteins was analyzed by Western blotting. Briefly, these proteins were probed with the following primary antibodies: mouse anti-CD86 (1:500), anti-iNOS (1:500), anti-CD163 (1:500), anti-IL-10 (1:500), anti-β-actin (1:1000), rabbit anti-PI3K (1:400), anti-Akt (1:400), or anti-p-Akt(Ser473) (1:400) (all from eBioscience and Abcam Inc.). The detailed procedure has been previously described (20, 28).

### 2.6 Flow cytometry studies

To analyze the macrophage phenotype, THP-1 cells were induced to differentiate into macrophages of various phenotypes, according to the method described above. The cells were washed twice with pre-cooled PBS, PBS was added, and an appropriate amount of ACCUTASE cell digestion solution was added. The cells were gently shaken to evenly cover the cell surface and then placed in a cell incubator. When the cells gradually fell off, 3 mL of fresh medium was added to blow the adherent cells, and the cell suspension was centrifuged at 1500 RPM/min for 10 min. THP-1 cells were centrifuged at 800 RPM/min for 5 min, without digestion. Next, 100 μL of fixation solution was added to 100 μL of the cell suspension by blowing, and the tube was mixed by pulsed vortexing and incubated for 30 min at room temperature in the dark. At the end of fixation, 2 mL of 1×membrane breaking solution was added and centrifuged for 5 min at 1500 RPM/min at room temperature. The supernatant was discarded, the cell precipitate was resuspended in 100 μL of 1×membrane breaking solution, and the corresponding flow antibody or the respective isotype control was added according to the manufacturer’s instructions. The cells were incubated at room temperature for 30 min in the dark, and the cell suspension was shaken every 10 min during the incubation period to allow for full reaction with the antibody. At the end of the incubation period, 2 mL of 1×membrane-breaking solution or pre-cooled PBS was added to each tube and centrifuged for 5 min at 1500 RPM/min at room temperature. Finally, the stained cells were resuspended in 300 μL of flow cytometry staining solution and transferred to a 5 mL flow cytometry tube with a cell filter for operation. The cell apoptosis analysis procedure was as follows: a noncontact co-culture system was established using a Transwell chamber (pore size:0.4 μm). Macrophages (approximately 1×10^6^ cells/well) were inoculated in the upper chamber and placed above the six-well plate of HCC cells (approximately 1×10^6^ cells/well) for 48 h. RPMI 1640 medium was added at 1.5 mL and 2.6 mL inside and outside the small chamber, respectively. The HCC cells were digested, centrifuged, and resuspended twice in pre-cooled PBS. The cell number was adjusted to 1×10^6^ cells/mL by adding 1×Annexin V binding buffer, and 100 μL of the cell suspension was placed into a 1.5 mL EP tube. The Annexin V-PE/7-AAD Apoptosis Detection Kit(Beijing Biolaibo Technology Co., LTD, Beijing, China) was used to detect cell apoptosis. Five microliters of PE annexin V and 5 μL of 7-AAD solution were added, and the solution was gently blown with a pipette gun, mixed, and incubated at room temperature in the dark for 15 min. At the end of the incubation, 400 μL of 1×Annexin V binding buffer was added and then transferred to a 5 mL flow tube with a filter screen for loading. The procedure for the analysis of phagocytosis of macrophages was as follows: THP-1 cells were stimulated into M0-type macrophages using PMA in a six-well plate, the supernatant was discarded, the cells were washed with sterile PBS, and serum-free medium was added for 6 h. Carboxylate-modified polystyrene latex beads were prepared as follows: 100 μL of polystyrene latex bead suspension was added to 10 mL of 1% BSA and incubated in a 37°C water bath for 30 min, ultrasonicated for 5 min, and then used as prepared. Analysis of macrophage phagocytosis: The serum-free medium in the six-well plate was replaced with complete medium, and a certain volume of latex bead suspension was added and incubated in the cell incubator for 3 h and 6 h, respectively, in the dark. The six-well plate was removed according to the set phagocytosis time, pre-cooled PBS was added, and the plate was washed several times to remove the latex beads that had not been phagocytosed. The macrophages that had phagocytosed latex beads in the six-well plate were digested and centrifuged, resuspended in flow cytometry staining solution, and transferred to a 5 mL flow tube with a cell filter. The phagocytic ability of macrophages was detected using flow cytometry, and the fluorescence intensity represented the relative number of latex beads were phagocytosed by macrophages.

### 2.7 Laser confocal microscopy

M0-type macrophages (2×10^5^ cells/well) were cultured and infected with AFP-expressing negative control lentivirus vector(M0-NC) or AFP-expressing lentivirus vector(M0-AFP). Then, the phagocytic experiment with polystyrene latex beads was carried out by laying plates in laser confocal microscope Petri dishes, as described above. The cells were treated with carboxylate-modified polystyrene latex beads for 3 or 6 h, and then the un-engulfed polystyrene latex beads were removed by washing several times with precooled PBS. The cells were treated with 400 μL 4% paraformaldehyde for 20 min and washed twice with PBS containing 0.1% Triton X-100. Actin-Tracker Green was diluted in the staining working solution with an immunofluorescence secondary antibody diluent, and 300 μL was added to each well and incubated at room temperature for 40 min in the dark. The cells were washed twice with PBS containing 0.1% Triton X-100 for 4 min each wash. Next, 300 μL of DAPI staining solution was added to each well, and the staining was shielded from light for 5 min. The samples were washed three times with PBS containing 0.1% Triton X-100, and polystyrene latex beads engulfed by macrophages were observed and photographed using a laser confocal microscope.

### 2.8 Cell migration assay

Macrophages in each group were digested with ACCUTASE cell digestion solution, and the number of cells was adjusted to 1×10^6^/mL using medium without serum. A Transwell chamber (pore size, 8 μm) was used, and 500 μL of fresh RPMI 1640 medium containing 20% FBS was added to each well in the lower chamber. Two hundred microliters of cell suspension were added to each upper chamber, and the cells were cultured for 72 h in a cell incubator. The cells in the upper layer of the Transwell chamber were gently wiped with a cotton ball, washed twice with PBS, and treated with 4% paraformaldehyde for 20 min. The fixed Transwell chamber was cleaned twice with PBS and stained with 0.1% crystal violet for 20 min. After staining, the residual staining solution was cleaned with PBS and the plate was allowed to dry. Images were captured under an inverted microscope, and five fields were randomly selected for each group to calculate the average value.

### 2.9 Healthy donor-derived monocytes were induced to polarize into macrophages

Healthy human(donors) (aged 22-26 years, four males and two females) were selected, and 50mL of blood was collected from each donor. Monocyte separation was performed as previously described (29, 30). Then PMA was added to the cell for 24h, and then the cells were treated with LPS+IFN-γor IL-4+IL-13 for 24h, respectively, to obtain M0-like macrophages, M1-like macrophages and M2-like macrophages.

### 2.10 Phagocytosis assay with tumor-derived AFP (tAFP)

A patient with liver cancer was recruited from the Department of Hepatobiliary Surgery, the First Affiliated Hospital of Hainan Medical College. The serum AFP concentration of the patient was >5000 ng/mL. 100 ml of blood was collected and the tumor-derived AFP(tAFP) was purified following a previously described procedure (31, 32). M1-like macrophages derived from THP-1 and M1- or M2-like macrophages derived from monocytes were treated with tAFP(final concentration 20μg/mL) for 24h. The cells were resuspended in 1 mL of PBS, and 1 μL of 5-(and -6)-carboxyfluorescein diacetate succinimidyl ester (CFSE). Laser confocal microscopy and intelligent living-cell high-throughput imaging analyzer were used to observe macrophages phagocytizing polystyrene latex beads or liver cancer cells(HLE cells). The following describes the experimental operation of observing M1-like macrophages that phagocytize HCC cells using an intelligent living-cell high-throughput imaging analyzer.

### 2.11 Intelligent living-cell high-throughput imaging analyzer studies

To improve the fluorescence expression effect of HCC cells infected with lentivirus, Bel 7402 cells (untreated or infected with the interference AFP-expressing vector) were digested and centrifuged for cell counting. The cells were resuspended in 1 mL of PBS, and 1 μL of CFSE storage solution was added for blowing and mixing. The cells were incubated for 30 min at room temperature and then washed three times with PBS. M1-type macrophages were digested and centrifuged for cell counting. The stained liver cancer cells were co-cultured with M1-type macrophages in a six-well plate at a ratio of 1:1.5, and placed in a cell incubator for 2 h until the cells adhered to the wall. The bright field channel of the intelligent living-cell high-throughput imaging analyzer and the RFP red fluorescence channel (to reduce the strong green fluorescence background) were used to dynamically track and image the cells in contact with the co-culture. Five different visual fields were randomly selected for each group and photographed every 30 minutes for 24 hours.

### 2.12 Statistical analysis

Each experiment was repeated more than three times. All experimental data were analyzed using the GraphPad Prism software (version 9.0). A *t*-test was used to compare the differences between two groups, the Mann Whitney *U*-test was applied to compare the differences in macrophage marker; one-way ANOVA was used to compare the differences between multiple groups, and the data are expressed as the mean ± standard deviation (x ± SD). Differences were considered statistically significant at *P* < 0.05.

## 3 Results

### 3.1 Morphological characteristics to identify THP-1 monocytes and M0-, M1- and M2-type macrophages

**Figure 1:**
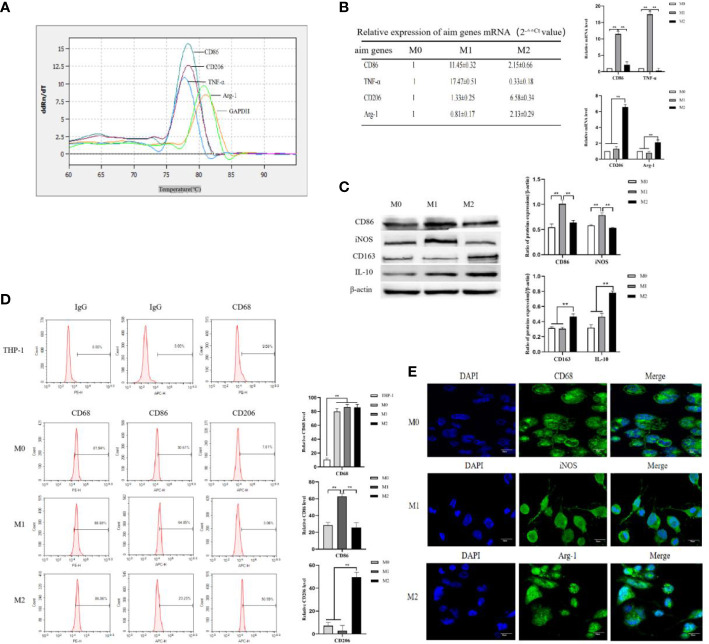
THP-1 monocytes were induced to polarize toward M0, M1 and M2 macrophages. **(A)** M0-type macrophages were induced to polarize toward M1- or M2-type macrophages using LPS+IFN-γ and IL-4+IL-13, respectively, for 72 h. Real-time PCR was used to detect the Ct values of the M1-type macrophage markers CD86 and TNF-α and the M2-type macrophage markers CD206 and Arg-1. Melting curve comparison of the relative mRNA expression levels of each target gene in M0, M1 and M2 macrophages. **(B)** The expression of each target gene in M0 macrophages was used as a control. The relative mRNA expression levels of each target gene in M1 and M2 macrophages were calculated by the ^2-△△^Ct method. The right column diagrams display the statistical differences in relative mRNA expression levels in each group, **P<0.01. **(C)** Protein expression of the M1-type macrophage markers CD86 and iNOS, and the M2-type macrophage markers CD163 and IL-10 detected by Western blotting; the right column diagrams display the expression levels of proteins in each group that were statistically analyzed by gray scanning, **P<0.01. **(D)** Expression of CD68 in the IgG isotype control and THP-1 cells; expression of CD68, CD86 and CD206 in M0, M1 and M2 macrophages detected by flow cytometry; the right column diagrams display statistical analysis of the expression of CD68, CD86 and CD206, **P<0.01. **(E)** The expression of the macrophage markers M0 (CD68), M1 (iNOS) and M2 (Agr-1) was observed by laser confocal microscopy. The results of the at least three independent experiments are shown.

THP-1 cells in good growth state were uniform in size, regular in shape, arranged in a circle under the microscope, and grown in suspension with clear cell boundaries and good refraction. After stimulation with RPMI 1640 medium containing 50 ng/mL PMA for 48 h, THP-1 cells gradually changed from suspension to adherent growth, lost their proliferative ability, increased in size, extended pseudopodia, and turned into tightly aligned oval or irregular M0-type macrophages. After stimulation of M0 macrophages with RPMI 1640 medium containing 50 ng/mL LPS+20 ng/mL IFN-γ for 24 h, the pseudopodia of M0-type macrophages changed more significantly, becoming M1-type macrophages with a long spindle shape. After stimulation of M0 macrophages with RPMI 1640 medium containing 20 ng/mL IL-4 + 20 ng/mL IL-13 for 72 h, the cell volume increased and the cells became polygonal M2-type macrophages (shown in **Supplementary materials: S-Figure 1**). The mRNA expression levels of macrophage markers were analyzed to identify the M1 and M2 types. M0 macrophages were induced to polarize toward M1- or M2-type macrophages using IFN-γ+LPS and IL-4+IL-13, respectively. Real-time PCR was used to detect the Ct values of the M1-type macrophage markers CD86 and TNF-α, and the M2-type macrophages markers CD206 and Arg-1. The expression of each target gene in the M0-type macrophages was used as a control. The relative mRNA expression levels of each target gene in the M1- and M2-type macrophages were calculated using the ^2-△△^Ct method. The experimental results showed that the melting curves of the reference gene GAPDH and the target genes CD86, TNF-α, CD206, and Arg-1 all had single peaks (**Figure 1A**), indicating that the amplification products had high specificity. The relative mRNA expression levels of CD86 and TNF-α in M1-type macrophages were higher than those in M0- and M2-type macrophages. The relative mRNA expression levels of CD206 and Arg-1 in M2-type macrophage were higher than those in M0- and M1-type macrophages (**Figure 1B**). These results indicated that CD86 and TNF-α were highly expressed in M1 macrophages upon stimulation with IFN-γ+LPS, but CD206 and Arg-1 were highly expressed in M2 macrophages upon stimulation with IL-4+IL-13. M0-type macrophages were induced to polarize toward M1- or M2-type macrophages using IFN-γ+LPS and IL-4+IL-13, respectively. The protein expression levels of the M1 macrophages markers CD86 and iNOS and the M2 macrophages markers CD163 and IL-10 were detected by Western blotting. The experimental results showed that the protein expression levels of CD86 and iNOS in M1 macrophages were higher than those in M0 and M2 macrophages, and the protein expression levels of CD163 and IL-10 in M2 macrophages were higher than those in M0 and M1 macrophages (**Figure 1C**). These results indicated that M1-type macrophages overexpressed CD86 and iNOS under the stimulation of IFN-γ+LPS, and that M2-type macrophages overexpressed CD163 and IL-10 under the stimulation of IL-4+IL-13.

CD68, as a 110-kDa highly glycosylated transmembrane protein, is the most specific and widely used marker for macrophages to distinguish monocytes from lymphocytes. The Flow cytometry results showed that the expression rates of CD68 in M0, M1, and M2 macrophages were 81.94 ± 4.34%, 88.69 ± 3.59% and 86.06 ± 4.41%, respectively (**Figure 1E**). The expression rate of CD68 in these macrophages was significantly higher than that in THP-1 cells (9.58 ± 2.25%) (**Figure 1E**), indicating that CD68 was highly expressed in M0, M1, and M2 macrophages. D86, a 60 kDa molecule expressed on antigen-presenting cells, belongs to the type I membrane protein of the immunoglobulin superfamily and is a biomarker of M1 macrophages. M0-type macrophages were induced to polarize toward M1- or M2-type macrophages using IFN-γ+LPS and IL-4+IL-13, respectively. Flow cytometry results showed that the expression rate of CD86 in M1 macrophages was 64.85 ± 3.39%, which was higher than that in M0 macrophages (30.61 ± 3.19%) and M2 macrophages (23.23 ± 5.73%) (**Figure 1D**), indicating that M0 macrophages highly expressed CD86 after IFN-γ+LPS stimulation. CD206, also known as the macrophage mannose receptor (MMR), is a 175 kDa type I single-chain transmembrane glycoprotein with a multilectin receptor structure, which is a biomarker of M2 macrophages. M0 macrophages were induced to polarize toward M1 or M2 macrophages using IFN-γ+LPS and IL-4+IL-13, respectively. Flow cytometry results showed that the expression rate of CD206 in M2 macrophages was 50.59 ± 4.25%, which was higher than that in M0 macrophages(7.61 ± 3.07%) and M1 macrophages(3.06 ± 4.48%) (**Figure 1D**), indicating that M0 macrophages highly expressed CD206 after stimulation with IL-4+IL-13. Laser confocal microscopy was used to observe the fluorescence expression of the M0-type macrophage marker CD68 (green fluorescence), the M1-type macrophage marker iNOS (green fluorescence), and the M2-type macrophage marker Arg-1 (green fluorescence). DAPI (blue fluorescence) staining was used to determine the location of nuclei. The merged images represent fluorescence expression on the surface of macrophages when the two types of fluorescence were fused (**Figure 1E**). The results showed that the pseudopodia of the M1 macrophages were more obvious than those of the M0 and M2 macrophages. CD68, iNOS, and Arg-1 were expressed in the M0, M1, and M2 macrophages, respectively.

### 3.2 Establishment of cell lines stably overexpressing or interfering with AFP expression, and the influence of AFP on the expression of macrophage markers

**Figure 2:**
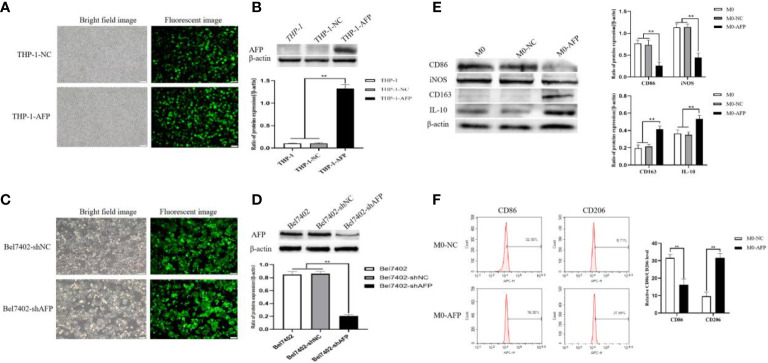
Establishment of stable overexpression of AFP in the THP-1-cell line and stable interference with AFP expression in the Bel7402 cell line, and AFP influences the expression of macrophage markers. **(A)** Fluorescence microscope was applied to observe the expression of GFP in THP-1 cells while infected with AFP-expressing negative control lentivirus vectors (M0-NC) or AFP-expressing lentivirus vectors (M0-AFP) after 48 h. **(B)** The expression of AFP was detected by Western blotting; the low column diagram shows statistical analysis of the expression levels of AFP in each group by gray scanning, **P<0.01. **(C)** Fluorescence microscope was applied to observe the expression of GFP in Bel7402 cells while infected with interference AFP-expressing vectors after 48 h. **(D)** The expression of AFP was detected by Western blotting; the low column diagram shows statistical analysis of the expression levels of AFP in each group by gray scanning, **P<0.01. **(E)** The expression of the M1-type macrophage markers CD86 and iNOS, and the M2-type macrophage markers CD163 and IL-10 was detected by Western blotting; the right column diagram displays the expression levels of macrophage markers in each group, which were statistically analyzed by gray scanning, **P<0.01. **(F)** Effect of AFP overexpression on the expression of the M1-type macrophage marker CD86, and the M2-type macrophage marker CD206 detected by flow cytometry; the right column diagram shows statistical analysis of the expression levels of CD86 and CD206, **P<0.01. The pictures are representative photos of three independent experiments.

THP-1 cells were infected with AFP-expressing lentivirus vectors and the expression of green fluorescent protein (GFP) in the cells was observed under an inverted fluorescence microscope for 72 h (**Figure 2A**). The GFP expression rate in THP-1 cells was approximately 80%. After puromycin screening, the culture was continued, and cellular proteins were extracted. The protein expression levels of AFP in the cells were detected by Western blotting (**Figure 2B**). The results showed that the protein expression of AFP in THP-1 cells while infected with AFP-expressing lentivirus vectors was significantly higher than that in the control group THP-1-NC (negative control, NC), proving that a THP-1-AFP cell line stably overexpressing AFP was successfully constructed. The Bel7402 cell line was infected with short hairpin RNA to suppress AFP(shAFP) lentiviral vectors, and the expression of green fluorescent protein (GFP) in the cells was observed under an inverted fluorescence microscope after 72 h (**Figure 2C**). The positive rate of GFP expression in the Bel7402 cells was approximately 90%. After puromycin screening, the culture was continued, and cell proteins were extracted. Western blotting was performed to detect the protein expression (**Figure 2D**). The results indicated that the protein expression of AFP in Bel7402-shAFP cells was significantly lower than that in the control group Bel7402-shNC (negative control, NC), proving that the Bel7402-shAFP cell line stably interfering with AFP expression was successfully established. THP-1 cells stably expressing AFP were stimulated with PMA to polarize them into M0 macrophages. Then, the cells were treated with LPS+IFN-γ or IL-4+IL-13, and the expression levels of the M1 macrophage markers CD86 and iNOS, and the M2 macrophage markers CD163 and IL-10 were detected by Western blotting. The results showed that the expression of CD163 and IL-10 in the macrophages of the M0-AFP (infected with AFP-expressing lentivirus vectors) group was higher than that in the M0 and M0-NC (infected with unexpressed lentivirus vectors) groups, and the protein expression levels of CD86 and iNOS in the macrophages of the M0-AFP group were lower than those in the M0 and M0-NC groups (**Figure 2E**). These results indicate that AFP overexpression in macrophages could upregulate the protein expression of the M2-type macrophage markers CD163 and IL-10 and downregulate the protein expression of the M1-type macrophage markers CD86 and iNOS. These results indicated that AFP overexpression promotes polarization of macrophages into the M2 type.

THP-1 cells stably expressing AFP were stimulated with PMA to polarize them into M0-type macrophages. M0-AFP cells were then treated with LPS+IFN-γ or IL-4+IL-13. Flow cytometry was used to detect the expression of the M1-type macrophage marker CD86 and M2-type macrophage marker CD206. The results showed that the expression rate of the M2-type macrophage marker CD206 in the M0-AFP group was 31.59 ± 1.99%, which was higher than that in the M0-NC group (9.71 ± 2.38%). The expression rate of the M1-type macrophage marker CD86 in macrophages of the M0-AFP group was 16.30 ± 2.04%, which was lower than that in the M0-NC group (32.30 ± 1.74%) (**Figure 2F**). These results indicated that AFP overexpression in macrophages could upregulate the expression of the M2-type macrophage marker CD206 and downregulate the expression of the M1-type macrophage marker CD86.

### 3.3 AFP activates the PI3K/Akt signaling pathway and stimulates macrophages to polarize into M2 type

**Figure 3:**
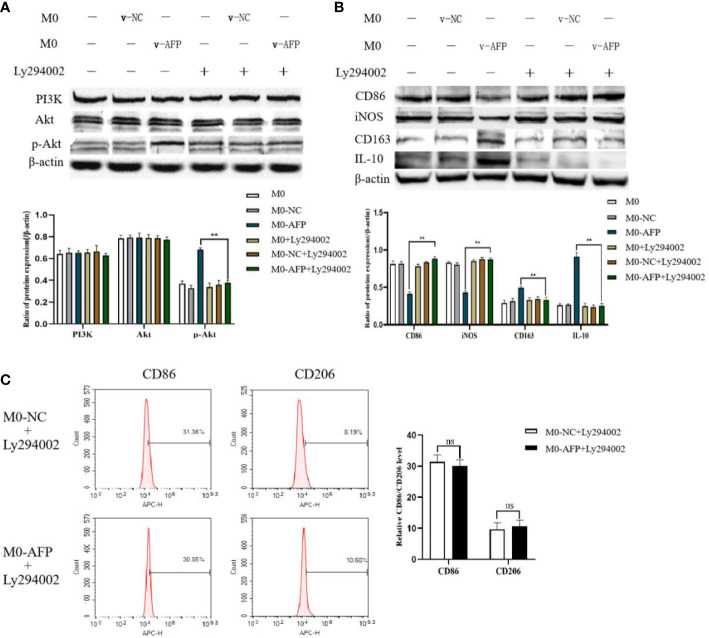
The effects of AFP and the PI3K inhibitor Ly294002 on the expression of M1- and M2-type macrophages’ markers. The M0 macrophages were infected with AFP-expressing negative control lentivirus vectors (M0-NC) or AFP-expressing lentivirus vectors (M0-AFP) for 48 h and then treated with LPS+IFN-γ or IL-4+IL-13 for 24 h, followed by treated with Ly294002 (final concentration: 20 μmol/L) for 24 h. **(A)** The expression of PI3K, Akt and p-Akt(Ser473) proteins in the M0-NC and M0-AFP groups was analyzed by Western blotting; the right column diagram displays the expression levels of proteins in each group statistically analyzed by gray scanning, **P<0.01; **(B)** The expression of the M1-type macrophage markers CD86 and iNOS and the M2-type macrophage markers CD163 and IL-10 in the M0-NC and M0-AFP groups analyzed by Western blotting; the right column diagram displays the expression levels of proteins in each group statistically analyzed by gray scanning, **P<0.01. **(C)** The expression of the M1-type macrophage marker CD86 and the M2-type macrophage marker CD206 in macrophages of the M0-NC and M0-AFP groups was detected by flow cytometry; the right column diagram displays statistical analysis of the expression of CD86 and CD206 in each group (NS: *P >*0.05). The results of the at least three independent experiments are shown. v-NC: negative control lentivirus vectors; v-AFP: AFP-expressing lentivirus vectors.

Changes in the expression of M1- and M2-type macrophage markers after treatment with the PI3K inhibitor Ly294002 were detected by Western blotting. To investigate whether the PI3K/Akt signaling pathway is involved in the effect of AFP on the macrophages phenotype, changes in the expression of PI3K, Akt, and p-Akt(Ser473) in macrophages of the M0-NC and M0-AFP groups (treated with LPS+IFN-γ or IL-4+IL-13, respectively) were detected by western blotting. The results showed that the expression of PI3K and Akt in macrophages of the M0-NC and M0-AFP groups did not change significantly, but the expression of p-Akt(Ser473) in macrophages of the M0-AFP group was higher than that in macrophages of the M0-NC group (**Figure 3A**). To further verify whether AFP regulates the PI3K/Akt signaling pathway and affects the macrophage phenotype, the PI3K/Akt pathway inhibitor Ly294002 was used to treat the cells for 24 h before PMA was used to induce THP-1 polarization into the above two groups of macrophages. Western blotting was used to detect changes in the protein expression of the M1-type macrophage markers CD86 and iNOS, and the M2-type macrophage markers CD163 and IL-10. The results indicated that, compared with the macrophages in the M0-AFP group without Ly294002 treatment, the expression of CD86 and iNOS significantly increased, but the expression of p-Akt(Ser473), CD163, and IL-10 significantly decreased when the cells were treated with Ly294002(**Figure 3B**). These results indicated that AFP could promote the expression of CD163 and IL-10 proteins in macrophages; treatment with the pathway inhibitor Ly294002 successfully blocked the PI3K/Akt signaling pathway and the effect of AFP on macrophage polarized phenotype was inhibited.

Changes in the M1 and M2 macrophages phenotype markers after treatment with Ly294002 were detected using flow cytometry. After PMA was used to induce THP-1 cells into macrophages in the M0-NC and M0-AFP groups, the PI3K/Akt pathway inhibitor Ly294002 was added and incubated for 24 h. Flow cytometry was used to detect the expression of the M1-type macrophage phenotype marker CD86 and the M2-type macrophage phenotype marker CD206. The flow cytometry results showed that the expression rate of the M1-type macrophages phenotype marker CD86 in the M0-NC group was 31.36 ± 3.29%, and there was no significant difference between the M0-NC and M0-AFP groups (30.85 ± 2.88%). The expression rate of the M2-type macrophage phenotype marker CD206 in the M0-NC group was 8.19 ± 2.48%, which was not significantly different from that in the M0-AFP group (10.60 ± 1.95%) (**Figure 3C**). The above results showed that after blocking the PI3K/Akt signaling pathway with Ly294002, there was no significant difference in the expression levels of CD86 and CD206 in the macrophages of the M0-AFP and M0-NC groups (*P >*0.05). These results indicated that the effect of AFP on the macrophage polarized phenotype was inhibited after blocking the PI3K/Akt signaling pathway, which proves that AFP promotes macrophage polarization toward the M2-type phenotype by activating the PI3K/Akt signaling pathway.

### 3.4 Overexpression of AFP in macrophages was able to enhance the migratory ability of macrophages and inhibit apoptosis of HCC cells.

**Figure 4:**
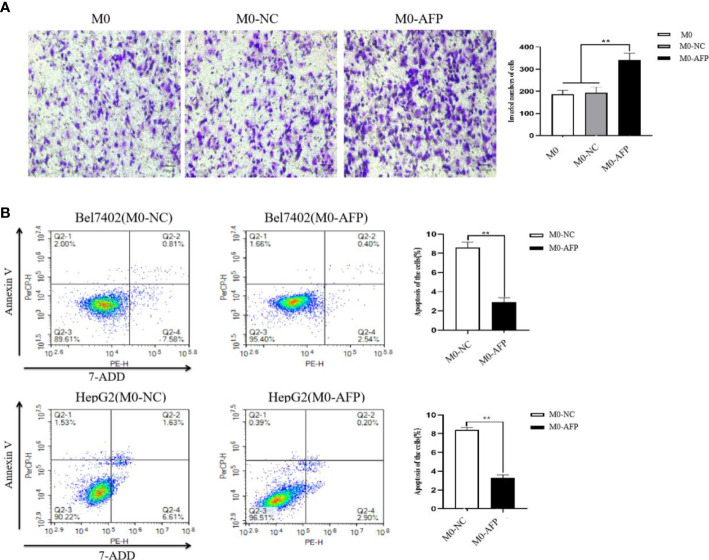
Effect of AFP on the migration of macrophages and apoptosis of HCC cells. M0 macrophages were infected with AFP-expressing negative control lentivirus vectors(M0-NC) or AFP-expressing lentivirus vectors(M0-AFP) for 48 h and then treated with LPS+IFN-γ for 24 h to induce M0 macrophage polarize into the M1-like phenotype. **(A)** The number of cells crossing the Transwell chamber membrane in each group was observed by inverted microscope after 72 h, and 5 fields were randomly selected to calculate the mean value of cells crossing the membrane; the right column diagram displays the statistical analysis of the number of invaded cells in each group, **P<0.01. **(B)** Bel7402 and HepG2 hepatoma cells were co-cultured with M0-NC or M0-AFP, and the effect of macrophages on apoptosis of HCC cells was detected by flow cytometry; the right column diagram displays the statistical analysis of the apoptosis rate of liver cancer cells, **P<0.01. The images are from the at least three independent experiments.

To detect the effect of AFP on the migratory ability of macrophages, the M0, M0-NC, and M0-AFP groups of macrophages were subjected to a 72 h migration assay in a Transwell chamber (pore size:8 μm). The results showed that the migratory numbers of macrophages in the M0-AFP group of macrophages were higher than those in the M0 and M0-NC groups (**Figure 4A**), indicating that AFP promoted the migratory ability of macrophages. The M0-NC and M0-AFP groups of macrophages were co-cultured with Bel7402 and HepG2 cells without contact in a Transwell chamber (pore size:0.4 μm) for 48 h, and apoptosis of HCC cells was detected by flow cytometry. The experimental results showed that the apoptosis rate of Bel7402 cells co-cultured with M0-AFP cells was 2.94 ± 0.48%, which was lower than that of Bel7402 cells co-cultured with M0-NC cells (8.39 ± 0.55%) (**Figure 4B**). The apoptosis rate of HepG2 cells co-cultured with M0-AFP group was 3.10 ± 0.33%, which was lower than that of HepG2 cells co-cultured with M0-NC group (8.24 ± 0.25%) (**Figure 4B**). These results indicated that AFP-overexpressing macrophages in the co-culture system could inhibit the apoptosis of HCC cells.

### 3.5 Macrophage-derived AFP inhibits macrophages to phagocytize polystyrene latex beads by activating the PI3K/Akt pathway

**Figure 5:**
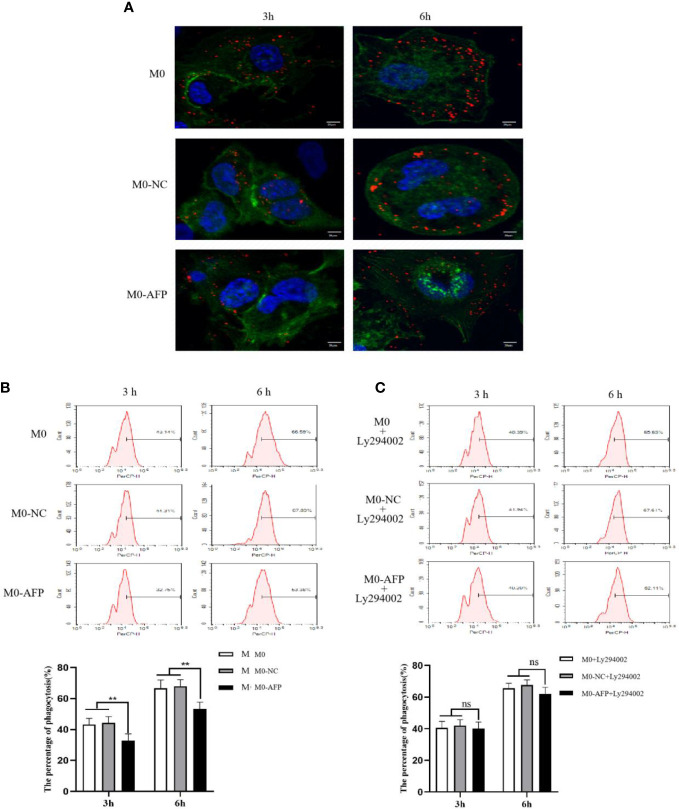
The effects of AFP and Ly294002 (PI3K inhibitor) on macrophages enggulfing polystyrene latex beads. M0 macrophages were infected with an AFP-expressing negative control lentivirus vector(M0-NC) or AFP-expressing lentivirus vector(M0-AFP) for 48 h and then treated with LPS+IFN-γ for 24 h to induce M0 macrophage polarization into M1-like phenotype. **(A)** Macrophages engulfed polystyrene latex beads in the M0, M0-NC and M0-AFP groups at 3 h and 6 h were observed by laser confocal microscopy; the fluorescence intensity represents the numbers of phagocytoses. Blue: cell nucleus(DAPI stained); Green: cytoplasm(5-(and -6)-carboxyfluorescein diacetate succinimidyl ester(CFSE) incorporation of the intracellular fluorescent dye); Red: polystyrene latex beads. **(B)** Macrophages engulfed the numbers of polystyrene latex beads in the M0, M0-NC and M0 AFP groups at 3 h and 6 h, detected by flow cytometry; the lower column diagram shows the statistical analysis of the phagocytosis rate of each group, **P<0.01. **(C)** After treatment with the PI3K inhibitor Ly294002 for 24 h, macrophages engulfed the numbers of polystyrene latex beads in the M0, M0-NC and M0-AFP groups at 3 and 6 h were detected by flow cytometry; the low column diagram shows the statistical analysis of the phagocytosis rate of each group, *P*>0.05. The images are from the at least three independent experiments.

The effects of AFP overexpression on macrophages engulfing polystyrene latex beads were observed using laser confocal microscopy. To study the effect of AFP on the phagocytosis of macrophages, the actin-Tracker Green-stained M0-NC and M0-AFP groups engulf carboxylate-modified polystyrene latex beads with a diameter of 0.5 μm (red fluorescence) was observed by laser confocal microscopy. The cell nucleus were stained with DAPI (blue fluorescence) staining (**Figure 5A**). The results showed that macrophages engulfed polystyrene latex beads were mainly located at the irregular membrane edge of the macrophage processes, and a few were located around the nucleus. In M0, M0-NC, and M0-AFP groups, macrophages engulfed significantly more polystyrene latex beads at 6 h than at 3 h. At the same phagocytosis time, macrophages engulfed the number of polystyrene latex beads in the M0 and M0-NC groups was higher than that in the M0-AFP group.

The effect of AFP overexpression on the macrophages engulfing polystyrene latex beads was detected by flow cytometry. To further verify the effect of AFP on the phagocytic ability of macrophages to engulf polystyrene latex beads, flow cytometry was used to detect the fluorescence intensity after M0, M0-NC, and M0-AFP macrophages engulfed polystyrene latex beads *in vivo.* The fluorescence intensity indicates the relative numbers of polystyrene latex beads in the macrophages; the stronger fluorescence intensity of the polystyrene latex beads, the larger the quantity. The results of flow cytometry showed that the macrophages phagocytosis rates at 3 h and 6 h in the M0-AFP group were 32.75 ± 3.39% and 53.38 ± 2.28%, respectively; in the M0 group, they were 43.14 ± 3.12% and 66.59 ± 3.36%, respectively; in the M0-NC group, they were 44.31 ± 2.98% and 67.89 ± 3.47%, respectively. The experimental results showed phagocytosis of macrophages in each group at 3 h and 6 h, and the numbers of polystyrene latex beads in macrophages of the M0-AFP group was significantly lower than that in macrophages of the M0 and M0-NC groups (**Figure 5B**), indicating that the phagocytic ability of macrophages engulfed with polystyrene latex beads was decreased in macrophages which overexpressing AFP.

After PMA was used to induce THP-1 to become macrophages in the M0-NC and M0-AFP groups, the cells were treated with the PI3K/Akt pathway inhibitor Ly294002 for 24 h. Macrophages engulfed polystyrene latex beads in the M0-NC, M0-NC, and M0-AFP groups were detected by flow cytometry at 3 and 6 h. The results showed that the macrophages phagocytosis rate in M0 groups were 40.39 ± 3.2% and 65.63 ± 4.15% at 3 and 6 h, respectively, and the macrophages phagocytosis rates in the M0-NC group were 41.94 ± 2.82% and 67.61 ± 3.37% at 3 and 6 h, respectively. The macrophages phagocytosis rates in the M0-AFP group were 40.20 ± 3.04% and 62.11 ± 2.4% at 3 h and 6 h, respectively (**Figure 5C**). Statistical analysis of the macrophages phagocytosis rates in each group showed no significant differences (*P*>0.05). Compared to that without Ly294002 treatment, the macrophage phagocytosis rate in the M0 and M0-NC groups did not change significantly, but the phagocytic ability of the M0-AFP group was enhanced, indicating that AFP could activate the PI3K/Akt signaling pathway to inhibit the phagocytic ability of macrophages to engulf polystyrene latex beads.

### 3.6 Inhibition of AFP expression could enhance the phagocytic effect of macrophages on HCC cells

**Figure 6:**
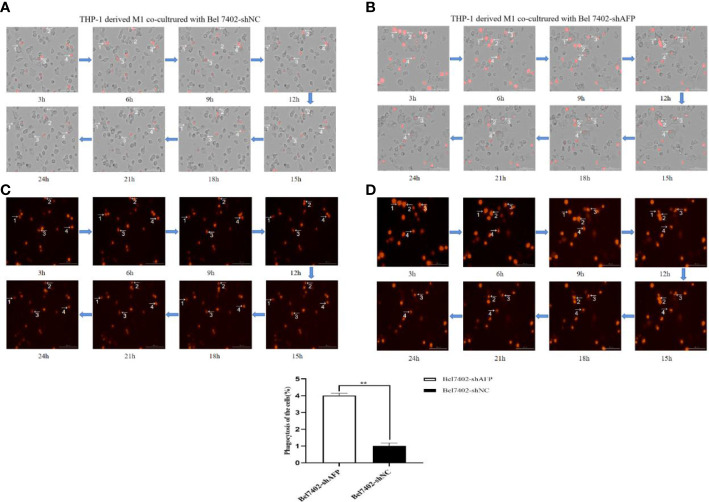
The effect of AFP on macrophages phagocytize HCC cells. M0 macrophages were administered LPS+IFN-γ for 24 h to induce M0 macrophage polarization into the M1-like phenotype. **(A)** A bright field channel was used to photograph the M1 macrophages co-cultured with Bel7402-shNC group. **(B)** A bright field channel was used to photograph the M1 macrophages co-cultured with Bel7402-shAFP group; **(C)** Bel7402-shNC cells were photographed using the RFP channel; **(D)**, Bel7402-shAFP cells were photographed using the RFP channel. The macrophages phagocytized HCC cells was observed by an intelligent living-cell high-throughput imaging analyzer, and images were taken every 30 min for 24 h. The low column diagram shows statistical analysis of the numbers of phagocytic cells in 5 randomly selected fields, **P<0.01. The images are from the at least three independent experiments. Red: Bel7402 cells; gray: THP-1 derived M1-type macrophages.

M1 macrophages can kill tumor cells and inhibit the growth of tumor cells through phagocytosis and Th1 response. To study the phagocytosis of M1 macrophages on HCC cells in the process of contact and co-culture with Bel7402-shAFP and Bel7402-shNC HCC cells, five different visual fields were randomly selected for the M1 macrophages co-cultured with Bel7402-shAFP group, and M1 macrophages co-cultured with Bel7402-shNC group using an intelligent living cell high-throughput imaging analyzer to conduct dynamic tracking and imaging. The morphology and position of M1 macrophages changed continuously during the contact and co-culture of the two cell types. Contact with Bel7402-shAFP cells, the morphology of M1 macrophages changed, the cell membrane invaginated, and Bel7402-shAFP cells were gradually phagocytosed and lysed (**Figures 6A, B**). When exposed to Bel7402-shNC cells, M1 macrophages only swam around the edges of Bel7402-shNC cells, without phagocytosis (**Figures 6C, D**). The dynamic video of M1 macrophage phagocytize HCC cells showed in attachment (**Supplementary Videos 1, 2**). These results indicated that the number of M1 macrophages that phagocytosed Bel7402-shAFP cells in the co-culture process was higher than that in the Bel7402-shNC group, proving that AFP expression in HCC cells may inhibit the phagocytic ability of macrophages.

### 3.7 Tumor-derived AFP(tAFP) inhibited macrophages to phagocytize polystyrene latex beads or HCC cells

**Figure 7:**
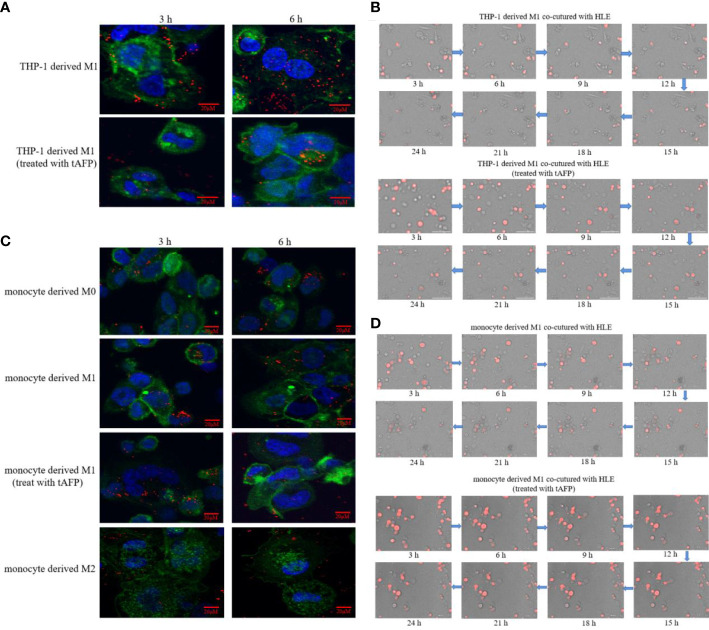
The effect of tAFP on macrophages phagocytizing polystyrene latex beads or HCC cells. Monocytes from health donors were treated with PAM to induce the polarization into M0 macrophage, then M0 macrophages were administered with LPS+IFN-γ or IL-4+IL-13 for 24 h to induce M0 macrophage polarize towards M1-like phenotype or M2-like phenotype(showed in **Supplementary materials: S-Figure 2**). The THP-1 derived M1-like macrophage(gray) or health monocytes derived macrophages(gray) co-cultured with polystyrene latex beads(red) or HLE cells(red), then treated with tAFP(final concentration 20μg/mL). **(A)** THP-1 derived M1-like macrophage co-culture with polystyrene latex beads and treated with tAFP, the images of macrophages phagocytize polystyrene latex beads were taken by laser laser confocal microscopy after co-culture for 3h or 6h. Blue: cell nucleus(DAPI stained); Green: cytoplasm(CFSE incorporation of the intracellular fluorescent dye); Red: polystyrene latex beads. **(B)** THP-1 derived M1-like macrophages co-cultured with HLE cells and treated with tAFP, the macrophages phagocytosis was observed by an intelligent living-cell high-throughput imaging analyzer, the bright field channel was used to photograph, and images were taken every 30 min for 24 h. **(C)** Health monocytes-derived macrophage co-cultured with polystyrene latex beads and treated with tAFP, the images of macrophages phagocytize polystyrene latex beads were taken by laser laser confocal microscopy after co-culture for 3h or 6h. **(D)** Health monocytes derived M1-like macrophages co-cultured with HLE cells, then treated with tAFP, the M1-like macrophages phagocytosis was observed by an intelligent living-cell high-throughput imaging analyzer, the bright field channel was used to photograph, and images were taken every 30 min for 24 h. The images are from the at least three independent experiments.

In order to observe the effect of AFP on macrophages phagocytizing polystyrene latex beads or HCC cells, tumor-derived AFP(tAFP) from liver cancer loading patient was purified, and healthy human(donor) monocytes were collected. First, we observed the influence of tAFP in THP-1 derived M1-like macrophages phagocytizing polystyrene latex beads or liver cancer cells(HLE(non-AFP expressed line)), the results indicated that when treated with tAFP(final concentration 20μg/mL), the THP-1 derived M1-like macrophages phagocytized polystyrene latex beads or HLE cells was significantly decreased (**Figures 7A, B**). Second, monocytes from healthy donors were collected and treated with PMA for 24 h, followed by stimulation with LPS+IFN-γ or IL-4+IL-13 for 24 h, Monocytes were then induced to polarize into M0, M1, and M2(shown in **Supplementary materials: S-Figure 2**); then, the polarized monocytes were co-cultured with polystyrene latex beads or HLE cells. The results showed that tAFP was able to significantly inhibit the monocytes derived M1-like macrophages to phagocytize polystyrene latex beads or HLE cells (**Figures 7C, D**); Moreover, an intelligent living-cell high-throughput imaging analyzer was used in this study to dynamically track observe the effect of tAFP on M1-like macrophages phagocytizing HLE cells, the dynamic video of M1-like macrophages phagocytize HLE cells shows in attachment (**Supplementary Videos 3, 4**). These results further proved that tAFP could significantly inhibit M1-like macrophages to phagocytize HCC cells.

## 4 Discussion

In early tumor microenvironment (TME) tissues, M1 macrophages, which inhibit angiogenesis and activate tumor immunity, are the main macrophage type. After tumor progression, the proportion of M2-type macrophages becomes the dominant type of infiltration in the TME, and M2-type macrophages can promote angiogenesis and inhibit immune response. In the early stages of tumor metastasis, macrophages are recruited from distant organs and improve the tissue microenvironment by secreting cytokines, thus providing a suitable microenvironment for tumor survival and distant metastasis (24). Owing to the plasticity of macrophages, the transformation of tumor-promoting M2 macrophages into tumor-killing M1 macrophages is closely associated with the prognosis of patients with liver cancer. Recently, Mantovani A, et al. (33) has suggested that macrophages could be used as an effective tool for targeted cancer therapy. As the first line of defense against exogenous invading pathogens, macrophages are an important bridge between the innate and adaptive immunity. Macrophages can recognize and phagocytose invading pathogens or cell debris in the form of fixed or free cells by expressing several receptors and secreting various bioactive substances. Macrophages can activate lymphocytes and other immune cells to kill pathogens (34, 35). Phagocytic receptors, such as Fc receptors(FcRs), exist on the membranes of macrophages and participate in phagocytic processes, they can be roughly divided into diverse types (36, 37). The study of the anti-tumor effects of macrophages has become a new focus in the exploration of tumor immunotherapy.

The phosphatidylinositol 3-kinase (PI3K)/Akt pathway has been reported to regulate cell growth, proliferation, differentiation, and metabolism, and its abnormal activation plays an important role in the occurrence and development of cancers, cardiovascular diseases, and diabetes (38–40). PI3K is a member of the intracellular lipid kinase family and can be divided into three classes according to its substrate specificity. Among them, class I, consisting of the catalytic subunit p110 (α,β,γ) and regulatory subunit P85, can be activated by signaling factors that promote the phosphorylation of PIP2 to PIP3 (41). PIP3 recruits Akt to bind to its PH domain with high affinity, inducing the phosphorylation of serine/threonine residues (Thr308/Ser473). Fully activated Akt can activate downstream signaling molecules to regulate cell function (42). The PI3K/Akt signaling pathway also plays an important role in the phenotypic polarization of macrophages, and activation of the PI3K/Akt pathway promotes the polarization of M2-type macrophages (43–45). As the most significant effector of PI3K, Akt comprises serine/threonine protein kinases (Akt1, Akt2, and Akt3) (46, 47). Knockdown of Akt1 can upregulate the expression of miRNA-155 and induce macrophage polarization toward the M1 type. Knockdown of Akt2 can upregulate the expression of miRNA-146a and induce polarization of macrophages into the M2 type (48, 49). THP-1 cells were isolated from the peripheral blood of a one-year-old boy with acute myelocytic leukemia(AML). THP-1 cells are convenient for stable subculture and cryopreservation *in vitro*. THP-1 cells are similar to human monocytes in terms of cell morphology, membrane surface antigens, and secreted cytokines; therefore, THP-1 cells are widely used in the study of related mechanisms, drug functions, and signaling pathways in monocytes and macrophages (50, 51). Therefore, THP-1 cells were used in the subsequent experiments. Lipopolysaccharide(LPS)+IFN-γ and IL-4+IL-13 were used according to the classic induction method, and the appropriate concentration was found to stimulate macrophages to polarize into M1- or M2- type (25, 52). THP-1 cells, stimulated by the differentiation inducer phorophester(PMA), activate the protein kinase C(PKC) pathway, stop proliferating, gradually turn from suspended cells to adherent cells, extend pseudopodia, generate phagocytic vesicles, and become macrophages with irregular nuclei (53). AFP is used as a specific index for the screening and diagnosis of primary liver cancer and has many important biological functions, including as a growth regulating factor with a two-way adjustment function, regulating the expression of oncogenes, promoting the growth and proliferation of tumor cells, invasion and metastasis of autophagy, inhibiting tumor cell apoptosis, suppressing the activation of T cells and DC cells, inducing apoptosis of these cells, and thus escaping immune surveillance, further causing malignant cancer transformation (54–56). At present, the relationship between AFP and macrophage polarization, and the influence of AFP on the TME has rarely been reported. To investigate whether AFP could affect the phenotype and phagocytic effect of macrophages, we established a THP-1 cell line stably overexpressing AFP by constructing an AFP-expressing lentiviral vector and stimulated it with PMA to polarize into M0 macrophages. After stable AFP expression in M0 macrophages, THP-1 cells were treated with IFN-γ+LPS or IL-4+IL-13. Flow cytometry and Western blotting were used to detect the expression of M1-type macrophage- and M2-type macrophage-related markers in M0 macrophages stably expressing AFP and to explore the relationship between changes in the macrophage phenotype and the PI3K/Akt signaling pathway. The effects of AFP overexpression on the migratory ability of macrophages and apoptosis rate of HCC cells in the co-cultured system were evaluated. The phagocytic function of macrophages is key to the body’s defense, immunity maintenance, and tissue homeostasis. To investigate whether AFP could inhibit the phagocytic ability of macrophages, we studied the effect of AFP overexpression on the phagocytic ability of macrophages. Macrophages phagocytize tumor cells is one of the anti-tumor mechanisms of macrophages, and M1 macrophages play an important role in the antigen presenting and phagocytizing tumor cells. In the present study, co-cultured M1 macrophages with Bel7402-shNC and Bel7402-shAFP cells, and an intelligent living-cell high-throughput imaging analyzer was used to dynamically track and image AFP-expressing cells to observe phagocytosis by macrophages. In-depth exploration of the relationship between AFP and phagocytosis of macrophages, and targeted intervention of AFP could provide new therapeutic hope for patients with liver cancer.

At present, the efficacy of surgical resection, transcatheter arterial chemoembolization, and radiofrequency ablation in the treatment of liver cancer is not satisfactory, and targeted therapy for liver cancer is an urgent problem to be solved (57, 58). With the development of tumor immunology, new immunotherapies have become a hot and challenging topic in basic cancer research. Immune checkpoint inhibitors targeting programmed death receptor-1(PD-1), programmed cell death ligand-1(PD-L1), cytotoxic T-lymphocyte-associated antigen-4 (CTLA-4), and chimeric antigen receptor T-cell immunotherapy(CAR-T) have been developed. Emerging immunotherapies such as these have been approved by the FDA and have entered the clinical application stage, they have been proven to have remarkable efficacy in the treatment of some advanced malignant tumors, and can prolong the overall survival of tumor patients compared with traditional treatment methods (59, 60). However, despite bringing new hope to cancer treatment, these immunotherapies still have certain limitations. Therefore, it is necessary to identify new therapeutic targets for immune intervention to develop more effective clinical immunotherapies for liver cancer (61, 62).

To investigate whether AFP could affect the macrophage phenotype, we constructed THP-1 cell lines stably expressing AFP and stimulated them with PMA to induce polarization into M0-type macrophages. Flow cytometry and Western blotting analysis showed that the M2-type macrophage markers CD163, CD206, and IL-10 were significantly expressed in AFP-overexpressing M0 macrophages, whereas the M1-type macrophage markers iNOS and CD86 were expressed at lower levels. It has been proven that AFP could promote macrophage polarization from M0 to M2. Evidence has been shown that the PI3K/Akt signaling pathway can regulate the phenotypic polarization of macrophages and thus affect the occurrence, development, and prognosis of tumors (63). In this study, we found that the effect of AFP on the macrophage phenotype was inhibited after treatment with Ly294002, an inhibitor of the PI3K/Akt signaling pathway, indicating that AFP promotes macrophage polarization toward the M2-type by activating the PI3K/Akt signaling pathway.

AFP, a G protein-coupled receptor (GPCRs) agonist, can regulate cell migration and invasion. Human recombinant AFP protein can increase the mRNA and protein levels of matrix metalloproteinase 9(MMP9), thereby enhancing the invasive ability of THP-1 cells in a concentration-dependent manner (26). To explore whether AFP could affect the migratory ability of THP-1-derived macrophages, we conducted Transwell chamber analyses and found that the migratory ability of M0 macrophages which overexpressing AFP was stronger than that of the control group, indicating that AFP overexpression could also enhance the migratory ability of THP-1-derived macrophages. M0 macrophages overexpressing AFP and macrophages in the control group were co-cultured with Bel7402 and HepG2 cells in a Transwell chamber (pore size:0.4 μm) without contact. Flow cytometry results showed that co-cultured with AFP-overexpressing macrophages reduced the apoptosis rate of Bel7402 and HepG2 cells. In this study, we found that AFP could promote the transformation of M0 macrophages into M2 macrophages. Therefore, it is speculated that the decreased apoptosis rate of HCC cells may be related to the secretion of pro-tumor cell growth factors by macrophages.

It has been reported that purified human recombinant AFP protein could inhibit the phagocytic ability of macrophages in chicken red blood cells by binding to mouse peritoneal macrophages, and this inhibition could be relieved to varying degrees after AFP removal (64). In this study, laser confocal microscopy and flow cytometry analysis indicated that the phagocytic ability of M0 macrophages which overexpressing AFP for polystyrene latex beads was significantly lower than that of the control group, indicating that AFP could inhibit the phagocytic ability of macrophages for polystyrene latex beads. Signal regulatory protein α (SIRPα) on the surface of macrophages can help macrophages recognize “myself” and “non-me” cells, and the surface of tumor cells usually highly express the CD47 molecule. CD47 can bind to SIRPα on the surface of macrophages to convey the “don’t eat me” signal to macrophages, forming the antiphagocytic signaling axis, inhibiting phagocytosis of macrophages on tumor cells (65, 66). In addition to CD47, the macrophage surface leukocyte immunoglobulin-like receptor B1(LILRB1) protein can specifically recognize major histocompatibility complex I(MHC I) and microglobulin-like β2 (β2M) on the surface of tumor cells, allowing tumor cells to directly escape macrophage phagocytosis (67). In a tumorigenesis experiment in nude mice, it was found that the survival time of nude mice was prolonged by nearly 70% after inhibiting the expression of MHC I on the surface of cancer cells. When the β-chains of CD47 and MHC I were simultaneously targeted, anti-tumor activity was significantly enhanced compared to that of CD47 or MHC I alone (68). Phagocytosis of macrophages in tumor cells is an anti-tumor mechanism. To study the differences in the phagocytic ability of macrophages in AFP-expressing HCC cells, we used an intelligent living-cell high-throughput imaging analyzer to contact and co-cultured M1 macrophages with Bel7402-shAFP and Bel7402-shNC cells and conducted dynamic tracking photography. M1 macrophages gradually phagocytized Bel7402-shAFP cells and lysed them by changing the cell morphology and position. When M1 macrophages came into contact with Bel7402-shNC cells, macrophages only swam around the edge of Bel7402-shNC cells without phagocytosis, indicating that AFP expression in HCC cells may inhibit macrophages to phagocytize hepatoma cells.

This study initially explored the effects of exogenous AFP overexpression in THP-1 cells on the macrophage phenotype, migration, and phagocytosis. Previous studies have reported that there is a specific AFP receptor on the cell membrane of THP-1 cells that may be involved in physiological regulation of the immune response (26, 69). Because immunotherapy of liver cancer is a hot scientific issue in research on the prevention and treatment of liver cancer, and AFP is a highly specific protein expressed by liver cancer cells, the immunosuppressive function of AFP has been widely studied (70), and the immunomodulatory function of AFP in hepatoma cells should be evaluated. To further study the effect of AFP on the phagocytosis of macrophages, in this study, liver cancer-derived AFP(tAFP) was treated in THP-1- derived M1 cells and monocyte-derived macrophages from healthy donors, and the effect of tAFP on macrophages phagocytizing, the phagocytosis of THP-1-derived M1 and monocytes-derived macrophages was observed. The results showed that tAFP not only inhibited THP-1-derived M1 cells phagocytizing polystyrene latex beads and HCC cells, but also inhibited monocytes-derived macrophages phagocytizing HCC cells. These results suggest that tAFP inhibits macrophages phagocytosis. In the future, normal AFP(nAFP) and tAFP proteins will be used to treat THP-1-derived macrophages and monocyte-derived macrophages from healthy donors, and to test the different effect of nAFP and tAFP on phagocytosis of the macrophage phenotype. In the present study, the results indicated that AFP has a novel function in stimulating M0-type macrophages polarization into M2-type macrophages and inhibiting M1-type macrophages to phagocytize HCC cells, implying that AFP plays a key role in anti-inflammation, accelerating HCC cells to escape from the attack of macrophages, and inhibiting adaptive anti-tumor immunity. AFP may be used in immunotherapy for patients with HCC.

AFP promotes polarization of M0 macrophages into M2-type and attenuates macrophages to phagocytize polystyrene latex beads. AFP also inhibited the ability of M1 macrophages to phagocytize HCC cells. The role of AFP in suppressing the phagocytic ability of M1 macrophages involves the activation of the PI3K/Akt signaling pathway. tAFP may be used as a novel biotarget for immunotherapy in HCC patients.

## Data availability statement

The original contributions presented in the study are included in the article/**Supplementary Material**. Further inquiries can be directed to the corresponding authors.

## Ethics statement

All experiments were approved by the committee of Hainan Medical College, Haikou, Hainan Province, China

## Author contributions

MZg, KL, QZ, and JX designed the experiments. JL, BL, and M Zg performed experiments. ML and MZu designed and supervised the study and analyzed the data. ML wrote the manuscript. All authors contributed to the article and approved the submitted version.

## Supplementary material

Supplementary Video 1 & 2 Observed dynamically track of AFP inhibits M1 phenotype macrophages phagocytize HCC cells was used an intelligent live-cell high-throughput imaging analyzer (video). M0 macrophages were treated with LPS +IFN-γ for 24 h, and M0 macrophages were induced to polarize into M1 phenotype macrophages. **(A)** M1 macrophages co-cultured with Bel7402-shNC group; images were taken every 30 min for 24 h. **(B)** M1 macrophages co-cultured with Bel7402-shAFP group; and images were taken every 30 min for 24 h. Red: Bel 7402 cells; gray: THP-1 derived M1 phenotype macrophages.

Supplementary Video 3 & 4 Dynamically track of tAFP inhibits healthy donor monocytes-derived M1-like phenotype macrophages phagocytize HLE cells was observed by an intelligent live-cell high-throughput imaging analyzer (video). **(A)** Health monocytes derived M1-like phenotype macrophages co-cultured with HLE group; and images were taken every 30 min for 24 h. **(B)** Health monocytes derived M1-like phenotype macrophages co-cultured with HLE, then treated with tAFP(final concentration 20μg/mL) group; and images were taken every 30 min for 24 h. Red: HLE cells; gray: M1-like phenotype macrophages.

## References

[1] Sung H Ferlay J Siegel RL Laversanne M Soerjomataram I Jemal A. Global cancer statistics 2020: GLOBOCAN estimates of incidence and mortality worldwide for 36 cancers in 185 countries. CA Cancer J Clin (2021) 71(3):209–49. doi: 10.3322/caac.21660 33538338

[2] Zhou W Zhang Q Qiao L. Pathogenesis of liver cirrhosis. World J Gastroenterol (2014) 20(23):7312–24. doi: 10.3748/wjg.v20.i23.7312 PMC4064077 24966602

[3] Rao H Wu E Fu S Yang M Feng B Lin A. The higher prevalence of truncal obesity and diabetes in American than Chinese patients with chronic hepatitis c might contribute to more rapid progression to advanced liver disease. Aliment Pharmacol Ther (2017) 46(8):731–40. doi: 10.1111/apt.14273 28833342

[4] Megahed FAK Zhou X Sun P. The interactions between HBV and the innate immunity of hepatocytes. Viruses (2020) 12(3):285. doi: 10.3390/v12030285 32151000 PMC7150781

[5] Galle PR Foerster F Kudo M Chan SL Llovet JM Qin S. Biology and significance of alpha-fetoprotein in hepatocellular carcinoma. Liver Int (2019) 39(12):2214–29. doi: 10.1111/liv.14223 31436873

[6] Trevisani F Garuti F Neri A. Alpha-fetoprotein for diagnosis, prognosis, and transplant selection. Semin Liver Dis (2019) 39(2):163–77. doi: 10.1055/s-0039-1677768 30849784

[7] Lopez-Yrigoyen M Cassetta L Pollard JW. Macrophage targeting in cancer. Ann N Y Acad Sci (2021) 1499(1):18–41. doi: 10.1111/nyas.14377 32445205

[8] Monnier M Paolini L Vinatier E Mantovani A Delneste Y Jeannin P. Antitumor strategies targeting macrophages: the importance of considering the differences in differentiation/polarization processes between human and mouse macrophages. J Immunother Cancer (2022) 10(10):e005560. doi: 10.1136/jitc-2022-005560 36270732 PMC9594518

[9] Szulc-Kielbik I Kielbik M. Tumor-associated macrophages: reasons to be cheerful, reasons to be fearful. Exp Suppl (2022) 113:107–40. doi: 10.1007/978-3-030-91311-3_4 35165862

[10] Bin-Zhi Q Jeffrey WP. Macrophage diversity enhances tumor progression and metastasis. Cell (2010) 141(1):39–51. doi: 10.1016/j.cell.2010.03.014 20371344 PMC4994190

[11] Wang H Yung MMH Ngan HYS Chan KKL Chan DW. The impact of the tumor microenvironment on macrophage polarization in cancer metastatic progression. Int J Mol Sci (2021) 22(12):6560. doi: 10.3390/ijms22126560 34207286 PMC8235734

[12] Zhu S Yi M Wu Y Dong B Wu K. Roles of tumor-associated macrophages in tumor progression: implications on therapeutic strategies. Exp Hematol Oncol (2021) 10(1):60. doi: 10.1186/s40164-021-00252-z 34965886 PMC8715617

[13] Scheurlen KM Snook DL Gardner SA Eichenberger MR Galandiuk S. Macrophage differentiation and polarization into an M2-like phenotype using a human monocyte-like THP-1 leukemia cell line. J Vis Exp (2021) 174). doi: 10.3791/62652 34398156

[14] Martinez FO Sica A Mantovani A Locati M. Macrophage activation and polarization. Front Biosci (2008) 13:453–61. doi: 10.2741/2692 17981560

[15] Aras S Zaidi MR. TAMeless traitors: macrophages in cancer progression and metastasis. Br J Cancer (2017) 117(11):1583–91. doi: 10.1038/bjc.2017.356 PMC5729447 29065107

[16] Deng L He K Pan Y Wang H Luo Y Xia Q. The role of tumor-associated macrophages in primary hepatocellular carcinoma and its related targeting therapy. Int J Med Sci (2021) 18(10):2109–16. doi: 10.7150/ijms.56003 PMC8040428 33859517

[17] Liu YC Zou XB Chai YF Yao YM. Macrophage polarization in inflammatory diseases. Int J Biol Sci (2014) 10(5):520–9. doi: 10.7150/ijbs.8879 PMC4046879 24910531

[18] Wang S Zhu M Wang Q Hou Y Li L Weng H. Alpha-fetoprotein inhibits autophagy to promote malignant behaviour in hepatocellular carcinoma cells by activating PI3K/AKT/mTOR signalling. Cell Death Dis (2018) 9(10):1027. doi: 10.1038/s41419-018-1036-5 30301886 PMC6177398

[19] Zhu M Li W Lu Y Dong X Lin B Chen Y. HBx drives alpha fetoprotein expression to promote initiation of liver cancer stem cells through activating PI3K/AKT signal pathway. Int J Cancer (2017) 140(6):1346–55. doi: 10.1002/ijc.30553 27925189

[20] Lu Y Zhu M Li W Lin B Dong X Chen Y. Alpha fetoprotein plays a critical role in promoting metastasis of hepatocellular carcinoma cells. J Cell Mol Med (2016) 20(3):549–58. doi: 10.1111/jcmm.12745 PMC4759472 26756858

[21] Ferrante CJ Pinhal-Enfield G Elson G Cronstein BN Hasko G Outram S. The adenosine-dependent angiogenic switch of macrophages to an M2-like phenotype is independent of interleukin-4 receptor alpha (IL-4Ra) signaling. Inflammation (2013) 36(4):921–31. doi: 10.1007/s10753-013-9621-3 PMC3710311 23504259

[22] Li MS Li PF Yang FY He SP Du GG Li G. The intracellular mechanism of alpha-fetoprotein promoting the proliferation of NIH 3T3 cells. Cell Res (2002) 12(2):151–6. doi: 10.1038/sj.cr.7290121 12118941

[23] Zhu M Guo J Li W Xia H Lu Y Dong X. HBx induced AFP receptor expressed to activate PI3K/AKT signal to promote expression of src in liver cells and hepatoma cells. BMC Cancer (2015) 15:362. doi: 10.1186/s12885-015-1384-9 25943101 PMC4427932

[24] Shao X Wu B Cheng L Li F Zhan Y Liu C. Distinct alterations of CD68+ CD163+ M2-like macrophages and myeloid-derived suppressor cells in newly diagnosed primary immune thrombocytopenia with or without CR after high-dose dexamethasone treatment. J Transl Med (2018) 16(1):48. doi: 10.1186/s12967-018-1424-8 29499727 PMC5833082

[25] Pinto SM Kim H Subbannayya Y Giambelluca MS Bösl K Ryan L. Comparative proteomic analysis reveals varying impact on immune responses in phorbol 12-Myristate-13-Acetate-mediated THP-1 monocyte-to-macrophage differentiation. Front Immunol (2021) 12:679458. doi: 10.3389/fimmu.2021.679458 34234780 PMC8255674

[26] Zubkova E Semenkova L Dudich E Dudich I Parfyonova Y Menshikov M. Alpha-fetoprotein contributes to THP-1 cell invasion and chemotaxis *via* protein kinase and gi-protein-dependent pathways. Mol Cell Biochem (2013) 379(1-2):283–93. doi: 10.1007/s11010-013-1650-6 23615710

[27] Hindson CM Chevillet JR Briggs HA Gallichotte EN Ruf IK Hindson BJ. Absolute quantification by droplet digital PCR versus analog real-time PCR. Nat Methods (2013) 10(10):1003–5. doi: 10.1038/nmeth.2633 PMC4118677 23995387

[28] Li M Li H Li C Wang S Jiang W Liu Z. Alpha-fetoprotein: a new member of intracellular signal molecules in regulation of the PI3K/AKT signaling in human hepatoma cell lines. Int J Cancer (2011) 128:524–32. doi: 10.1002/ijc.25373 20473866

[29] Nielsen MC Andersen MN Møller HJ. Monocyte isolation techniques significantly impact the phenotype of both isolated monocytes and derived macrophages in vitro. Immunology (2020) 159(1):63–74. doi: 10.1111/imm.13125 31573680 PMC6904589

[30] Kowalewicz-Kulbat M Ograczyk E Krawczyk K Rudnicka W Fol M. Type of monocyte immunomagnetic separation affects the morphology of monocyte-derived dendritic cells, as investigated by scanning electron microscopy. J Immunol Methods (2016) 439:79–82. doi: 10.1016/j.jim.2016.10.004 27746164

[31] Keel BA Eddy KB He Y Gangrade BK May JV. Purified human alpha-fetoprotein inhibits follicle-stimulating hormone-stimulated estradiol production by porcine granulosa cells in culture. Mol Cell Endocrinol (1993) 94(1):21–5. doi: 10.1016/0303-7207(93)90047-n 7690723

[32] Lin B Peng G Feng H Li W Dong X Chen Y. Purification and characterization of a bioactive alpha-fetoprotein produced by HEK-293 cells. Protein Expr Purif (2017) 136:1–6. doi: 10.1016/j.pep.2017.05.008 28554567

[33] Mantovani A Allavena P Marchesi F Garlanda C. Macrophages as tools and targets in cancer therapy. Nat Rev Drug Discovery (2022) 21(11):799–820. doi: 10.1038/s41573-022-00520-5 35974096 PMC9380983

[34] DeNardo DG Ruffell B. Macrophages as regulators of tumour immunity and immunotherapy. Nat Rev Immunol (2019) 19(6):369–82. doi: 10.1038/s41577-019-0127-6 PMC7339861 30718830

[35] Hallett MB. An introduction to phagocytosis. Adv Exp Med Biol (2020) 1246:1–7. doi: 10.1007/978-3-030-40406-2_1 32399822

[36] Rosales C Uribe-Querol E. Phagocytosis: A fundamental process in immunity. BioMed Res Int (2017) 2017:9042851. doi: 10.1155/2017/9042851 28691037 PMC5485277

[37] Lancaster CE Ho CY Hipolito VEB Botelho RJ Terebiznik MR. Phagocytosis: What’s on the menu? Biochem Cell Biol (2018) 97(1):21–9. doi: 10.1139/bcb-2018-0008 29791809

[38] Fruman DA Chiu H Hopkins BD Bagrodia S Cantley LC Abraham RT. The PI3K pathway in human disease. Cell (2017) 170(4):605–35. doi: 10.1016/j.cell.2017.07.029 PMC5726441 28802037

[39] Tewari D Patni P Bishayee A Sah AN Bishayee A. Natural products targeting the PI3K-Akt-mTOR signaling pathway in cancer: A novel therapeutic strategy. Semin Cancer Biol (2022) 80:1–17. doi: 10.1016/j.semcancer.2019.12.008 31866476

[40] Funk CR Wang S Chen KZ Waller A Sharma A Edgar CL. PI3Kδ/γ inhibition promotes human CART cell epigenetic and metabolic reprogramming to enhance antitumor cytotoxicity. Blood (2022) 139(4):523–37. doi: 10.1182/blood.2021011597 PMC8796652 35084470

[41] Rathinaswamy MK Burke JE. Class I phosphoinositide 3-kinase (PI3K) regulatory subunits and their roles in signaling and disease. Adv Biol Regul (2020) 75:100657. doi: 10.1016/j.jbior.2019.100657 31611073

[42] Linton MF Moslehi JJ Babaev VR. Akt signaling in macrophage polarization, survival, and atherosclerosis. Int J Mol Sci (2019) 20(11):2703. doi: 10.3390/ijms20112703 31159424 PMC6600269

[43] Zhao S Mi Y Guan B Zheng B Wei P Gu Y. Tumor-derived exosomal miR-934 induces macrophage M2 polarization to promote liver metastasis of colorectal cancer. J Hematol Oncol (2020) 13(1):156. doi: 10.1186/s13045-020-00991-2 33213490 PMC7678301

[44] Zhao SJ Kong FQ Jie J Li Q Liu H Xu AD. Macrophage MSR1 promotes BMSC osteogenic differentiation and M2-like polarization by activating PI3K/AKT/GSK3β/β-catenin pathway. Theranostics (2020) 10(1):17–35. doi: 10.7150/thno.36930 31903103 PMC6929615

[45] Lu Y Liu S Yang P Kou Y Li C Liu H. Exendin-4 and eldecalcitol synergistically promote osteogenic differentiation of bone marrow mesenchymal stem cells through M2 macrophages polarization via PI3K/AKT pathway. Stem Cell Res Ther (2022) 13(1):113. doi: 10.1186/s13287-022-02800-8 35313964 PMC8935829

[46] Ediriweera MK Tennekoon KH Samarakoon SR. Role of the PI3K/AKT/mTOR signaling pathway in ovarian cancer: Biological and therapeutic significance. Semin Cancer Biol (2019) 59:147–60. doi: 10.1016/j.semcancer.2019.05.012 31128298

[47] Arranz A Doxaki C Vergadi E Martinez de la Torre Y Vaporidi K Lagoudaki ED. Akt1 and Akt2 protein kinases differentially contribute to macrophage polarization. Proc Natl Acad Sci U.S.A. (2012) 109(24):9517–22. doi: 10.1073/pnas.1119038109 PMC3386059 22647600

[48] Wu X Chen H Wang Y Gu Y. Akt2 affects periodontal inflammation *via* altering the M1/M2 ratio. J Dent Res (2020) 99(5):577–87. doi: 10.1177/0022034520910127 32228353

[49] Feng Y Zheng C Zhang Y Xing C Cai W Li R. Triptolide inhibits preformed fibril-induced microglial activation by targeting the microRNA155-5p/SHIP1 pathway. Oxid Med Cell Longev (2019) 2019:6527638. doi: 10.1155/2019/6527638 31182996 PMC6512043

[50] Wasaporn C Jurriaan JM Harry JW. THP-1 cell line: An *in vitro* cell model for immune modulation approach. Int Immunopharmacol (2014) 23(1):37–45. doi: 10.1016/j.intimp.2014.08.002 25130606

[51] Park EK Jung HS Yang HI Yoo MC Kim C Kim KS. Correction to: Optimized THP-1 differentiation is required for the detection of responses to weak stimuli. Inflammation Res (2020) 69(11):1157. doi: 10.1007/s00011-020-01395-1 32918568

[52] Li P Hao Z Wu J Ma C Xu Y Li J. Comparative proteomic analysis of polarized human THP-1 and mouse RAW264.7 macrophages. Front Immunol (2021) 12:700009. doi: 10.3389/fimmu.2021.700009 34267761 PMC8276023

[53] Makino J Kamiya T Hara H Adachi T. TPA induces the expression of EC-SOD in human monocytic THP-1 cells: involvement of PKC, MEK/ERK and NOX-derived ROS. Free Radic Res (2012) 46(5):637–44. doi: 10.3109/10715762.2012.664841 22313459

[54] Munson PV Adamik J Butterfield LH. Immunomodulatory impact of α-fetoprotein. Trends Immunol (2022) 43(6):438–48. doi: 10.1016/j.it.2022.04.001 35550875

[55] Zhao K Zhou X Xiao Y Wang Y Wen L. Research progress in alpha-fetoprotein-induced immunosuppression of liver cancer. Mini Rev Med Chem (2022) 22(17):2237–43. doi: 10.2174/1389557522666220218124816 35184712

[56] Semeniuk DJ Boismenu R Tam J Weissenhofer W Murgita RA. Evidence that immunosuppression is an intrinsic property of the alpha-fetoprotein molecule. Adv Exp Med Biol (1995) 383:255–69. doi: 10.1007/978-1-4615-1891-4_27 8644510

[57] Sun J Yin T Zhang X Liu XJ. Therapeutic advances for patients with intermediate hepatocellular carcinoma. J Cell Physiol (2019) 234(8):12116–21. doi: 10.1002/jcp.28019 PMC13484215 30648254

[58] Sun HC Zhu XD. Downstaging conversion therapy in patients with initially unresectable advanced hepatocellular carcinoma: An overview. Front Oncol (2021) 11:772195. doi: 10.3389/fonc.2021.772195 34869008 PMC8636437

[59] Yu S Wang Y He P Shao B Liu F Xiang Z. Effective combinations of immunotherapy and radiotherapy for cancer treatment. Front Oncol (2022) 12:809304. doi: 10.3389/fonc.2022.809304 35198442 PMC8858950

[60] Posey AD Jr Schwab RD Boesteanu AC Steentoft C Mandel U Engels B. Engineered CAR T cells targeting the cancer-associated tn-glycoform of the membrane mucin MUC1 control adenocarcinoma. Immunity (2016) 44(6):1444–54. doi: 10.1016/j.immuni.2016.05.014 PMC5358667 27332733

[61] Kristian MH Coleman EJ Corey JW. Immune checkpoint blockade therapy for cancer: An overview of FDA-approved immune checkpoint inhibitors. Int Immunopharmacol (2018) 62:29–39. doi: 10.1016/j.intimp.2018.06.001 29990692

[62] El-Khoueiry AB Sangro B Yau T Crocenzi TS Kudo M Hsu C. Nivolumab in patients with advanced hepatocellular carcinoma (CheckMate 040): an open-label, non-comparative, phase 1/2 dose escalation and expansion trial. Lancet (2017) 389(10088):2492–502. doi: 10.1016/S0140-6736(17)31046-2 PMC7539326 28434648

[63] Lin F Yin HB Li XY Zhu GM He WY Gou X. Bladder cancer cell-secreted exosomal miR-21 activates the PI3K/AKT pathway in macrophages to promote cancer progression. Int J Oncol (2020) 56(1):151–64. doi: 10.3892/ijo.2019.4933 PMC6910194 31814034

[64] Lu CY Changelian PS Unanue ER. Alpha-fetoprotien inhibits macrophage expression of ia antigens. J Immunol (1984) 132(4):1722–7. doi: 10.4049/jimmunol.132.4.1722 6199409

[65] Gholamin S Mitra SS Feroze AH Liu J Kahn SA Zhang M. Disrupting the CD47-SIRPalpha anti-phagocytic axis by a humanized anti-CD47 antibody is an efficacious treatment for malignant pediatric brain tumors. Sci Transl Med (2017) 9(381):2968. doi: 10.1126/scitranslmed.aaf2968 28298418

[66] Logtenberg MEW Jansen JHM Raaben M Toebes M Franke K Brandsma AM. Glutaminyl cyclase is an enzymatic modifier of the CD47- SIRPα axis and a target for cancer immunotherapy. Nat Med (2019) 25(4):612–9. doi: 10.1038/s41591-019-0356-z PMC7025889 30833751

[67] Koller BH Marrack P Kappler JW Smithies O. Normal development of mice deficient in beta 2M, MHC class I proteins, and CD8+ T cells. Science (1990) 248(4960):1227–30. doi: 10.1126/science.2112266 2112266

[68] Barkal AA Weiskopf K Kao KS Gordon SR Rosental B Yiu YY. Engagement of MHC class I by the inhibitory receptor LILRB1 suppresses macrophages and is a target of cancer immunotherapy. Nat Immunol (2018) 19(1):76–84. doi: 10.1038/s41590-017-0004-z 29180808 PMC5832354

[69] Suzuki Y Zeng CQ Alpert E. Isolation and partial characterization of a specific alpha-fetoprotein receptor on human monocytes. J Clin Invest (1992) 90(4):1530–6. doi: 10.1172/JCI116021 PMC443200 1383274

[70] Sangro B Sarobe P Hervás-Stubbs S Melero I. Advances in immunotherapy for hepatocellular carcinoma. Nat Rev Gastroenterol Hepatol (2021) 18(8):525–43. doi: 10.1038/s41575-021-00438-0 PMC8042636 33850328

