# Supplementary material for: Alpha fetoprotein promotes polarization of macrophages towards M2-like phenotype and inhibits macrophages to phagocytize hepatoma cells
Source: Front Immunol. 2023 Feb 23;14:1081572. doi: 10.3389/fimmu.2023.1081572 (PMC9995430; doi:10.3389/fimmu.2023.1081572)
Supplement: Supplementary file 1 [file DataSheet_1.docx]

[Supplementary](javascript:;) [materials](javascript:;)


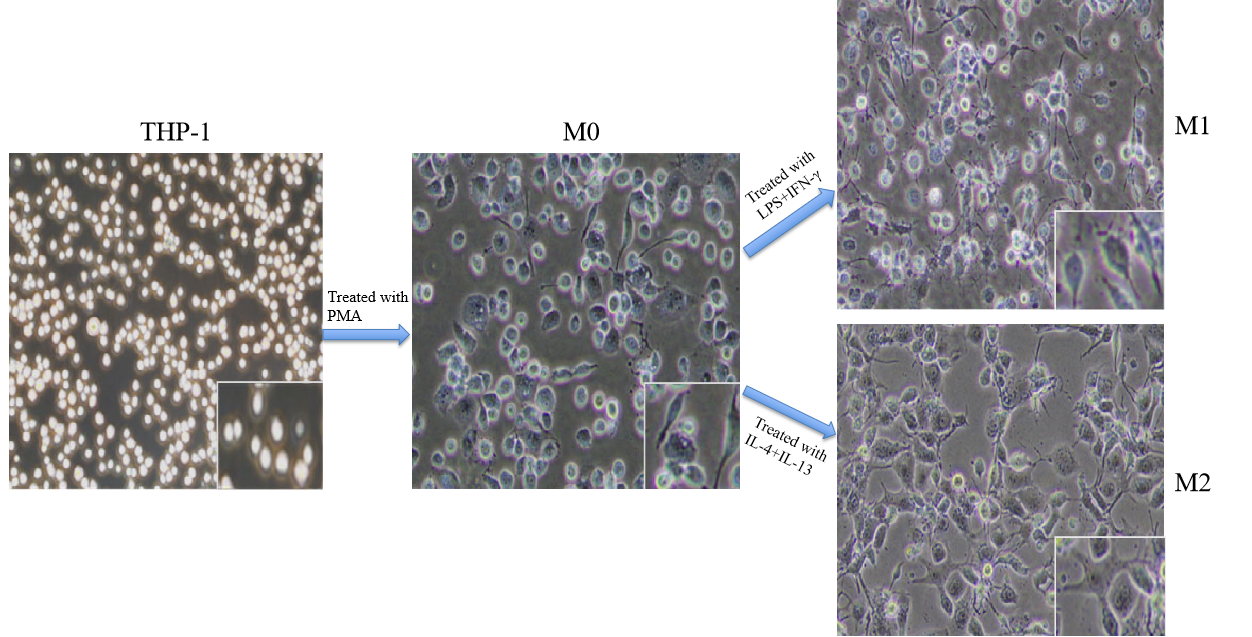


**s-Figure 1. THP-1 monocytes were induced to polarize towards M0, M1 and M2 macrophages**. Microscopy was applied to observe the morphological characteristics of THP-1 monocytes and M0, M1 and M2 macrophages. THP-1 cells were stimulated with RPMI 1640 medium containing 50 ng/mL PMA for 48 h. After THP-1 monocytes turned into M0 macrophages, the M0 macrophages were stimulated with RPMI 1640 medium containing 50 ng/mL LPS+20 ng/mL IFN-γ and 20 ng/mL IL-4+20 ng/mL IL-13 for 72 h. The morphological characteristics of these cells were observed by microscopy.


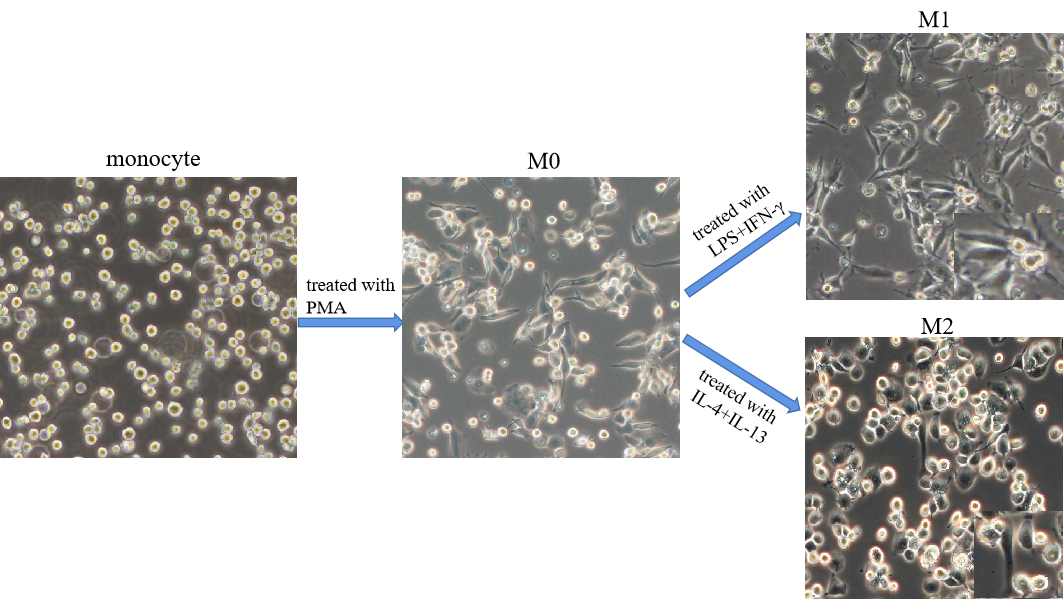


**s-Figure 2. Monocytes from health donors were induced to polarize towards M0, M1 and M2 macrophages**. Monocytes were treated with PAM to induce polarized into M0 macrophage, then M0 macrophages were administered LPS+IFN-γ or IL-4+ IL-13 for 24 h to induce M0 macrophage polarization into M1-like phenotype or M2-like phenotype. Microscopy was applied to observe the morphological characteristics of monocytes from health donors and M0, M1 and M2 macrophages.
